# Global Research Trends and Hotspots in Cardiac Devices: A Bibliometric and Visual Analysis

**DOI:** 10.3390/healthcare13233144

**Published:** 2025-12-02

**Authors:** Mohammed D. Al Shubbar, Raghad A. Alhojailan, Saeed A. Alzahrani, Assal Hobani, Hadeel H. Alabdulqader, Abdulrahman A. Alharbi, Sultan A. Alotibi, Norah S. Almuzil, Abdullah Al Jama

**Affiliations:** 1 Department of Internal Medicine, College of Medicine, Imam Abdulrahman Bin Faisal University, King Fahad Hospital of the University, Dammam 31441, Saudi Arabia; asjama@iau.edu.sa; 2 College of Medicine, Qassim University, Buraydah 52571, Saudi Arabia; raghadaziz90@gmail.com; 3 Department of Internal Medicine, King Fahad Armed Forces Hospital, Jeddah 23311, Saudi Arabia; alzahrani.a.saeed@gmail.com; 4 Faculty of Medicine, Ibn Sina National College for Medical Studies, Jeddah 22421, Saudi Arabia; assal7obani@gmail.com; 5 Department of Internal Medicine, King Abdulaziz University Hospital, Jeddah 22252, Saudi Arabia; hdlhamzah@gmail.com; 6 Family Medicine Academy, Madinah Health Cluster, Madinah 42313, Saudi Arabia; aaa.alharbi097@gmail.com; 7 Department of Internal Medicine, East Jeddah General Hospital, Jeddah 22253, Saudi Arabia; sultanahmedx1@gmail.com; 8 College of Medicine, University of Jeddah, Jeddah 23218, Saudi Arabia; nourasaleh0077@gmail.com

**Keywords:** pacemakers, leadless pacemaker, Cardiac implantable electronic devices, implantable cardioverter-defibrillator, Cardiac resynchronization therapy, self-powered pacemaker, bibliometric analysis

## Abstract

**Background**: Cardiac implantable electronic devices (CIEDs) have become indispensable tools in the management of bradyarrhythmia and heart failure, prompting a surge in research activity. To characterize the evolving research landscape, we conducted a bibliometric analysis focused on institutional contributions, author networks, journal trends, funding patterns, and emerging thematic hotspots in the field of cardiac devices to highlight keywords and identify knowledge development timelines and emerging trends, providing a comprehensive overview of the current state of research in this area. **Methods**: We conducted a bibliometric analysis of cardiac devices using the Web of Science Core Collection (WOSCC) on 27 November 2024, with search terms “ST (cardiac defibrillator) OR (pacemaker)”. Data from 1 January 2019 to 1 January 2024 resulted in 3753 articles, refined to 1000 after excluding non-English and methodologically inappropriate papers. VosViewer, Excel, and Drawio facilitated data visualization, creating networks where node size indicates frequency, line thickness shows association strength, and colors denote clusters. This approach helped identify key research trends and collaborations in the field. **Results**: The United States led in publication volume (362 papers) and citations (7198), with Emory University emerging as the most prolific institution. *Heart Rhythm* was the most productive journal, while *Europace* was the most co-cited. Kurt Stromberg was the leading author by publications and citations. Funding was predominantly from U.S. agencies, with the NIH and HHS each supporting 127 studies. Co-citation and keyword analyses revealed three dominant research clusters: (1) leadless pacemakers; (2) permanent pacemaker implantation following transcatheter aortic valve replacement (TAVR); and (3) development of self-powered pacing technologies, including piezoelectric and bioresorbable systems. **Conclusions**: This study offers a comprehensive overview of recent trends and intellectual structures in cardiac device research. By identifying key contributors, collaborative networks, and thematic evolutions, it provides a valuable reference for researchers, clinicians, and innovators seeking to navigate or shape the rapidly advancing field of cardiac electrophysiology and device therapy.

## 1. Introduction

The advent of cardiac devices has significantly transformed the field of cardiology by enabling therapeutic interventions aimed at stabilizing and treating bradyarrhythmias and enabling resynchronization [1]. These devices, depending on their type, have demonstrated considerable benefits in patients with heart failure and arrhythmias, including improvements in cardiopulmonary exercise capacity and substantial survival advantages [2].

As the availability of such devices has expanded, their use has risen dramatically, leading to increased numbers of patients benefiting from their therapeutic effects [3].

Cardiac devices range from pacemakers, which treat bradycardia and enable asymptomatic ventricular stimulation, to implantable cardioverter-defibrillators (ICDs) that are designed to prevent potentially fatal ventricular arrhythmias through shock delivery [4]. In parallel, bibliometrics, a method that applies statistical and mathematical tools to study the distribution and quantitative relationships within scientific literature, has gained prominence since its formal recognition in 1958 [5]. This approach has become an invaluable tool for assessing trends and providing insights in various medical fields, including cardiomyopathy, cardiorenal syndrome, pulmonary hypertension, chronic heart failure, and cardio-oncology [6]. Given the growing prevalence of cardiac devices in clinical practice over the past few decades, there has been a corresponding rise in the volume of publications on this subject. In light of this, we conducted a bibliometric review to study institutional characteristics, journal trends, author contributions, and citation patterns within the literature related to cardiac devices that were collected from Web of Science Core Collection (WoSCC). This study aims to highlight keywords and identify knowledge development timelines and emerging trends, providing a comprehensive overview of the current state of research in this area.

## 2. Materials and Methods

### 2.1. Search Strategies and Data Acquisitions

To perform a biometric analysis and visualize the latest research hotspots of cardiac devices, we conducted our search on 27 November 2024, using the Web of Science Core Collection (WOSCC). A topic search (TS) was performed using the Boolean string TS = (“cardiac defibrillator” OR “pacemaker”). This syntax retrieves records by searching the title, abstract, author keywords, and Keywords Plus fields simultaneously. Additionally, we utilized VosViewer V1.6.20, Excel (Microsoft 365), and Draw.io (https://www.drawio.com) software for effective data visualization and analysis. The dataset covered a five-year period from 1 January 2019 to 1 January 2024, resulting in a total of 3753 retrieved articles. After applying our exclusion criteria, we removed 66 articles that were not in English and 1570 with inappropriate methodologies. After screening 2117 eligible papers, we ranked them by total citation count and included the top 1000 most-cited records, without applying any numerical citation cut-off. This standardized approach ensured the inclusion of the most influential publications while maintaining analytical feasibility.

### 2.2. Bibliometric Analysis

Using bibliographic data from WOSCCS and Google Sheets, the data was analyzed to create visual representations. This process facilitated the development of a bibliometric network that helps researchers in the same field identify the most relevant keywords. Visualization consists of nodes and lines, where the thickness of the lines indicates the strength of associations. The size of the nodes represents the number of citations of a certain publication and the larger the size of the node, the higher the number of citations, while the colors denote the same cluster. Additionally, the overall length of the lines measures the level of collaboration. In this context, VOSviewer calculates a metric called Total Link Strength (TLS), which reflects the cumulative strength of all co-occurrence or co-authorship links connected to a given item (e.g., a keyword, author, or institution). A higher TLS value indicates that the item has stronger or more numerous connections within the network, thus signifying greater relevance or centrality. By applying this information from the visualization analysis, we can highlight keywords and identify knowledge development timelines and emerging trends, providing clearer insights into the developmental traits of researchers.

## 3. Results

### 3.1. An Overall Literature Findings

**Figure 1:**
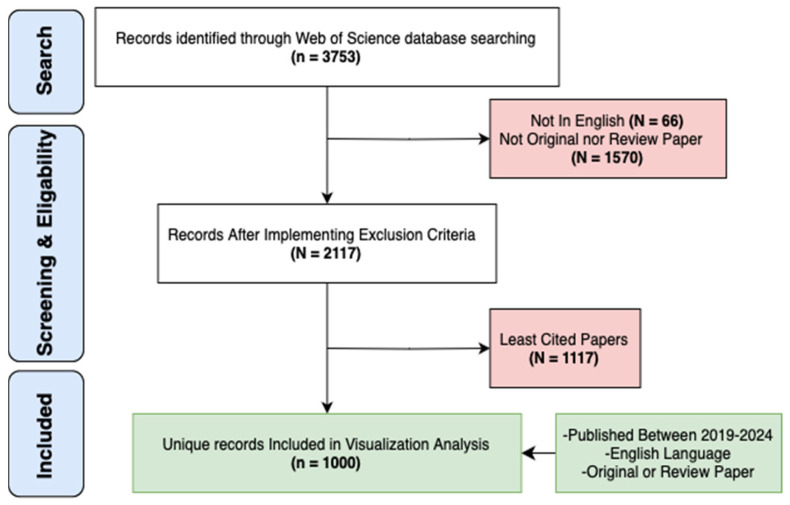
Flowchart of The Search Strategy.

For detailed illustration of the characteristics of desired publications, see (Figure 1).

### 3.2. The Regional Distribution of Global Publications Output

A total of 1071 publications related to cardiac devices were identified from the top ten contributing countries (Table 1). The United States dominated the field with 362 publications, accounting for approximately 33.8% of the global output. It also received the highest number of citations (7198) and demonstrated the strongest international collaboration, as indicated by its total link strength of 414. This suggests not only high research productivity but also strong integration within the global cardiac device research network.

Germany and Italy ranked second and third in publication volume, with 128 and 111 papers, respectively, and both showed high total citations and link strengths, reflecting both research quality and collaborative engagement. China, while ranking fourth in total publications -103-, had the lowest total link strength -82- and a relatively modest citation-per-publication ratio, suggesting a less influential role in international networks and possibly lower research impact per study. In contrast, England, the Netherlands, and France produced fewer publications but exhibited higher citation efficiency and collaborative strength, indicating that their contributions were proportionally more impactful.

Japan and Spain each contributed 66 publications, but Spain outperformed Japan in both citations and total link strength. Canada, though ranking tenth in output with 59 publications, maintained a moderate citation profile and collaborative presence.

**Figure 2:**
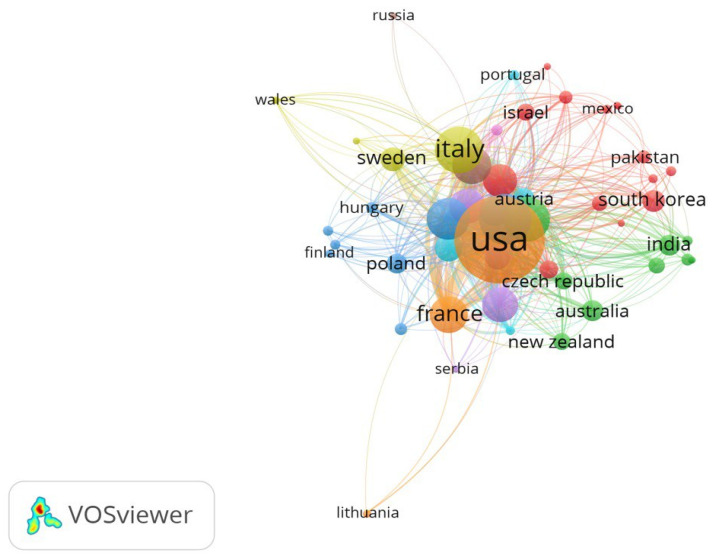
Network Visualization Map of Countries.

**Figure 3:**
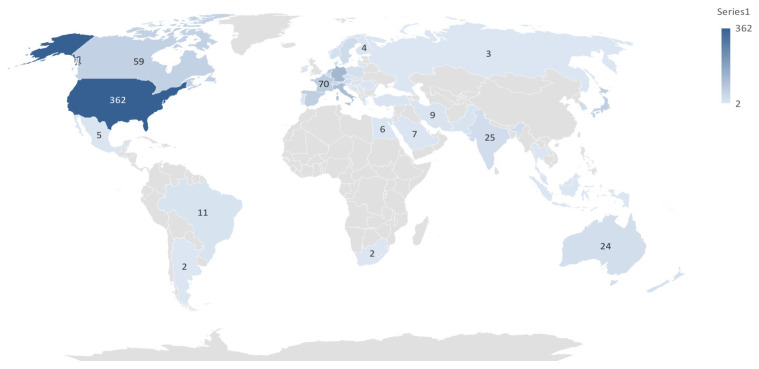
Graphical Distribution of Publication.

These geographical trends are further illustrated in Figure 2, which maps the density and strength of inter-country collaborations in cardiac device research. Additionally, Figure 3 provides a world map visualization of the global distribution of publications, further highlighting regional research hubs and global disparities in output.

### 3.3. Analysis of Institutional Output

A total of 174 publications on cardiac devices were contributed by the top 10 institutions (Table 2), with a notable concentration of output among U.S. academic centers. Emory University led in both volume (29 publications, 16.66%) and influence, evidenced by its highest citation-per-publication ratio (4.78) and top total link strength (TLS 451). This underscores its role as both a prolific and highly connected institution within the field. Mayo Clinic and Duke University followed in volume, but Duke’s higher citation rate (3.80) and strong network connectivity (TLS 373) suggest greater research influence relative to output.

Interestingly, Karolinska Institute and University of Amsterdam, though tied in rank with 18–19 publications, had substantially lower total link strengths (TLS 37 and 113, respectively), implying more localized or insular collaborations. Meanwhile, Duke Clinical Research Institute, with only 14 publications, ranked third in TLS (334) and had a moderate citation impact (2.71), reflecting its embeddedness in collaborative research networks despite lower output. At the other end of the spectrum, University of Leipzig had one of the lowest citation efficiencies (0.77) and link strengths (TLS 33), suggesting limited research visibility and integration.

**Figure 4:**
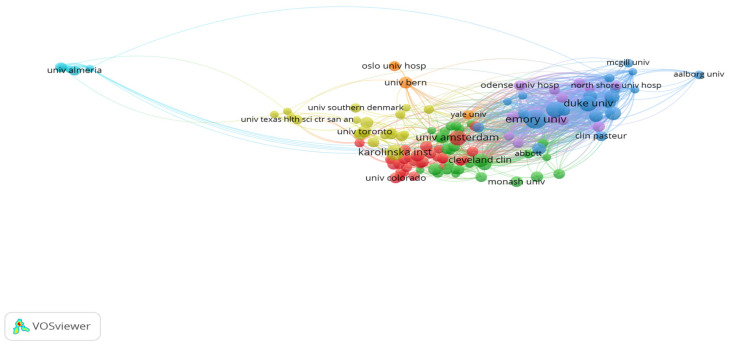
Network Visualization Map of Institutions.

These patterns are further illustrated in Figure 4, where institutions with higher TLS occupy more central nodes in the collaboration network. The dataset, analyzed using VOSviewer, included 130 nodes, 7 clusters, and 1166 inter-institutional links, revealing a core-periphery structure dominated by a few high-output, high-impact U.S. institutions.

### 3.4. Analysis of Journals and Co-Cited Journals

The analysis of the most productive and most co-cited journals (Table 3) reveals a clear concentration of research activity and influence within a small set of high-impact sources When ranked by the number of publications, Heart Rhythm emerged as the most prolific journal, contributing 53 articles, which accounts for 19.20% of all included publications. It was followed closely by the Journal of Cardiovascular Electrophysiology (51 articles, 18.47%) and PACE—Pacing and Clinical Electrophysiology (40 articles, 14.49%). Together, these three journals contributed over 50% of the total output in the field, underscoring their central role in disseminating original research on cardiac devices. Other moderate contributors include Europace (39 articles, 14.13%) and the Journal of Interventional Cardiac Electrophysiology (23 articles, 8.33%).

In contrast, when journals are ranked by total citations—reflecting co-citation influence—Europace takes the lead with 891 citations and a high citation-per-publication ratio of 22.84, despite being fourth in output. Heart Rhythm, which led in publication volume, ranks second in citations (820) and maintains a strong average of 15.47 citations per article. A notable outlier is JACC–Cardiovascular Interventions, which ranks third in citations (655) while publishing relatively fewer papers, resulting in the highest citation-per-publication ratio (59.54) among all top journals—highlighting the high impact of its individual articles. Similarly, European Heart Journal (48.14) and JACC–Clinical Electrophysiology (28.42) also demonstrate high per-publication influence, albeit with lower output.

These results demonstrate a dual dynamic in the field: a few journals dominate in publication volume, while others—particularly high-impact cardiology journals—achieve substantial citation density despite limited output. The total link strength (TLS) values further reflect these trends in the citation network. Journals such as Heart Rhythm (TLS: 132), Journal of Cardiovascular Electrophysiology (TLS: 108), and PACE (TLS: 81) not only publish frequently but also serve as highly interconnected nodes in the co-citation network.

This fused analysis highlights how both publication productivity and co-citation impact are concentrated within a select group of journals, reaffirming their status as essential platforms for advancing research and innovation in cardiac electrophysiology and device-based therapy.

**Figure 5:**
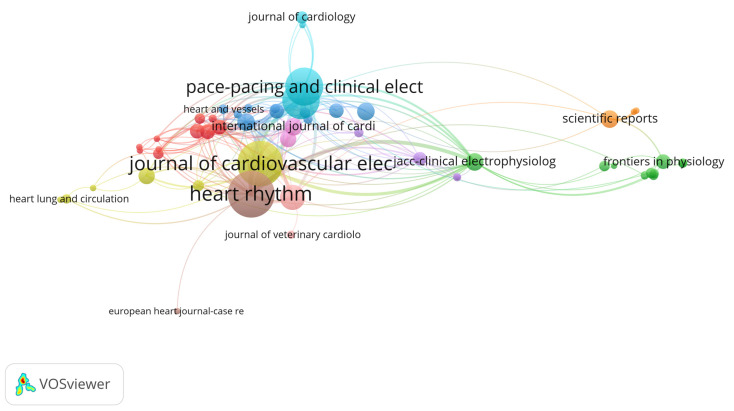
Network Visualization Map of Co-cited Journals.

The co-citation relationships among the most influential journals in the field are illustrated in the network visualization map (Figure 5).

### 3.5. Analysis of Authors and Co-Cited Authors

As shown in Table 4, author-level analysis revealed a strong overlap between research productivity and citation impact within the field of cardiac device research. Out of a total of 6591 authors, Kurt Stromberg emerged as the most prolific and most influential author, topping both the publication count (20 articles, 0.75%) and total citations (693), and recording the highest total link strength (TLS: 1273)—indicating a dominant position in both authorship and co-citation networks. Similarly, Mikhael F. El-Chami and Jonathan P. Piccini ranked second and third in productivity (18 and 17 publications, respectively) and were also among the top six most co-cited authors, with high TLS values (1074 and 1096, respectively), underscoring their central roles in shaping the discourse on cardiac devices.

Other notable figures include Christophe Garweg, Paul R. Roberts, and Laurence M. Epstein, who featured prominently in both productivity and citation influence, reflecting balanced contributions to the field. Conversely, authors such as Ning Li, Hao Zhang, and Ye Ma ranked highly in co-citation frequency despite not appearing among the top 10 publishing authors, suggesting that their work, while more limited in volume, has been foundational or frequently referenced across multiple studies. This discrepancy highlights the difference between volume-based influence and intellectual impact, with some researchers contributing high-impact publications that resonate widely across the literature.

**Figure 6:**
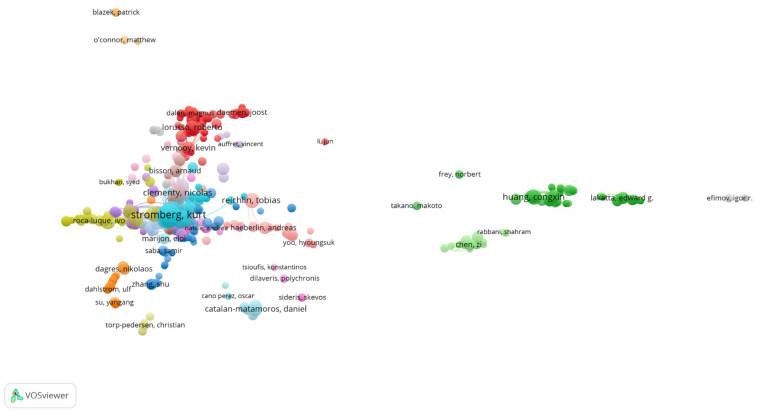
Network Visualization Map of Co-cited authors.

To further illustrate citation-based relationships, a co-citation network was constructed using VOSviewer, incorporating 991 authors who had published at least two articles. The resulting map, shown in Figure 6, visually represents the structural connectivity of the author network. The top three positions in total link strength were occupied by Kurt Stromberg (TLS: 1273, Citations: 693), Jonathan P. Piccini (TLS: 1096, Citations: 569), and Mikhael F. El-Chami (TLS: 1074, Citations: 487)—affirming their position as central figures in the collaborative and intellectual architecture of the field.

Overall, the author network demonstrates a concentration of academic influence among a small group of contributors, as reflected by the cumulative total link strength of 6498 across the top 10 authors. This indicates a tightly knit intellectual structure in the field, with a few key figures serving as both prolific authors and frequently cited thought leaders.

### 3.6. Analysis of Funding Agencies

Funding institutions serve as a critical driver of scientific progress by providing the financial support necessary to enable technological innovation and large-scale research efforts. As shown in Table 5, the most prominent funding source in the field of cardiac devices was the National Institutes of Health (NIH, USA), acknowledged in 127 articles, representing 23.69% of all funded studies. Interestingly, the United States Department of Health and Human Services (HHS) was cited in an equal number of publications (127 articles, 23.69%), reflecting overlapping or joint support between federal agencies.

The National Natural Science Foundation of China (NSFC) ranked third, supporting 66 articles (12.31%), highlighting China’s growing investment in cardiac device research. Industry funding was also notable, with Medtronic and Boston Scientific supporting 49 (9.14%) and 29 (5.41%) publications, respectively—underscoring the important role of private-sector funding in advancing device innovation and clinical translation.

Other significant contributors included the NIH’s National Heart, Lung, and Blood Institute (NHLBI) (35 articles, 6.53%) and major non-profit organizations such as the American Heart Association (25 articles, 4.66%). International support was also evident from agencies such as the German Research Foundation (DFG) and two Japanese funders: the Japan Society for the Promotion of Science and the Ministry of Education, Culture, Sports, Science and Technology (MEXT), each contributing to 25 publications (4.66%).

Overall, the funding landscape demonstrates a heavy reliance on U.S.-based federal and private institutions, which together account for over 70% of the top ten funding acknowledgments. However, the presence of strong international and industry participation highlights the global and multidisciplinary nature of cardiac device research.

### 3.7. Analysis of References

**Figure 7:**
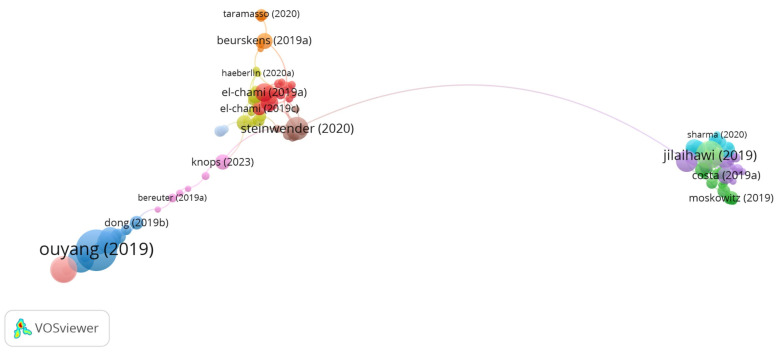
Network Visualization Map of Co-cited References [7,8,13,21,27,29,38,41].

Table 6 lists the 35 most-cited references in the field of cardiac devices, ranked by citation count. While Figure 7 presents the co-citation network map of references. The most cited study was authored by Ouyang et al. [7], titled “Symbiotic cardiac pacemaker,” with 446 citations, followed by “Minimizing Permanent Pacemaker Following Repositionable Self-Expanding Transcatheter Aortic Valve Replacement” by Jilaihawi et al. [8], cited 203 times. A majority of the highly cited studies were published in 2019 and appeared in Q1 journals, reflecting both the recency and high impact of foundational literature in this domain.

While citation count indicates the visibility and influence of individual studies, total link strength (TLS) reflects how frequently a reference is co-cited alongside others, thereby capturing its structural role within the scientific discourse. For example, “Costa (2019a)” [27], despite ranking 21st in citation count with 72 citations, recorded the highest TLS (13), highlighting its central position in the co-citation network. Similarly, “Sammour (2021a)” [15] and “Steinwender (2020)” [13] demonstrated high TLS values relative to their citation counts, suggesting their pivotal role in connecting related studies within the field.

Notably, several authors appear multiple times within the top 35 references, indicating consistent and influential contributions across multiple publications. The co-citation mapping revealed three distinct thematic clusters that represent the primary research foci in the field. The first cluster is centered on leadless pacemaker utility, particularly in the context of device extraction. The second focuses on permanent pacemaker implantation following transcatheter aortic valve replacement. The third encompasses the development of self-powered pacemaker technologies, particularly those employing piezoelectric nanogenerators.

Together, these findings demonstrate that the intellectual structure of cardiac device research is shaped by a small number of frequently cited studies that serve as key reference points across clinical, technical, and translational domains. The strong co-citation linkages observed in the network reflect a well-integrated and rapidly evolving field.

### 3.8. Analysis of Keywords and Keyword Co-Occurrence Clusters

**Figure 8:**
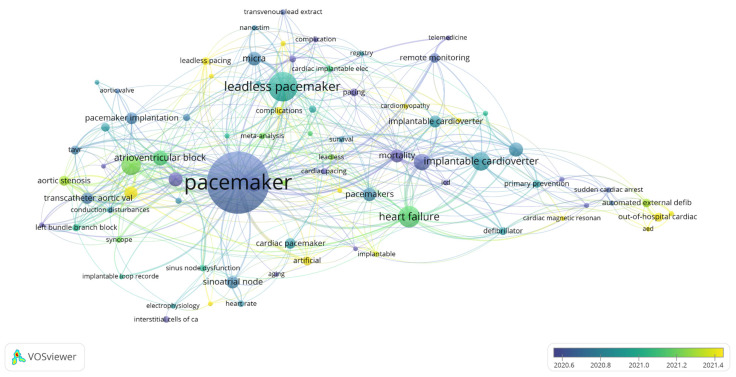
Cluster Analysis of Keywords Co-occurrence.

A total of 2232 keywords were identified across the included publications. For the co-occurrence analysis, 79 keywords that appeared at least seven times were selected. Figure 8 illustrates the network visualization map of these co-occurring keywords, which are distributed across seven distinct thematic clusters.

The keyword co-occurrence analysis offers a powerful tool for identifying research hotspots and emerging themes in the field of cardiac devices. High-frequency keywords enable both researchers and non-specialists to quickly locate relevant topics during literature searches and thematic reviews. As presented in Table 7, the most frequently occurring keywords include “pacemaker” (205 occurrences), “leadless pacemaker” (77 occurrences), “heart failure” (51 occurrences), “transcatheter aortic valve replacement” (45 occurrences), and “implantable cardioverter-defibrillator” (42 occurrences), among others. These terms represent core areas of investigation and clinical application.

As shown in Figure 8, the keywords are grouped into seven distinct thematic clusters, each representing a subtheme of research within the field of cardiac devices:

Cluster 1: Implantable Cardioverter-Defibrillator (42 occurrences, Total Link Strength [TLS] = 73)

Cluster 2: Transcatheter Aortic Valve Replacement (45 occurrences, TLS = 81)

Cluster 3: Leadless Pacemaker (77 occurrences, TLS = 92)

Cluster 4: Pacemaker (205 occurrences, TLS = 243)

Cluster 5: Atrial Fibrillation (31 occurrences, TLS = 50)

Cluster 6: Remote Monitoring (18 occurrences, TLS = 31)

Cluster 7: Syncope (11 occurrences, TLS = 22)

The clinical and scholarly relevance of the keyword co-occurrence map lies in its ability to group interrelated terms, offering a macro-level view of thematic directions in the literature. Each cluster encapsulates a set of interconnected concepts, enabling researchers to quickly identify subfields of interest, track thematic evolution, and align their work with both established and emerging trends in cardiac device research.

## 4. Discussion

### 4.1. The Most-Contributing Authors and the Most-Cited Papers

Cardiac implantable electronic devices (CIEDs) have revolutionized modern cardiology since their first clinical use in 1958 [42]. They now play a central role in the management of arrhythmias and heart failure, offering life-saving interventions that restore and maintain normal cardiac rhythms [1]. This bibliometric analysis provides a comprehensive overview of the research landscape, highlighting key authors, journals, and influential studies that have shaped the field of cardiac devices.

Between 2019 and 2024, the number of publications on cardiac devices increased markedly, with the United States emerging as the most prolific contributor, largely through academic–industry collaborations. The dominance of the United States reflects a confluence of sustained federal funding robust academic–industry partnerships, and the presence of specialized electrophysiology societies that foster collaboration. While this leadership accelerates device innovation, it also highlights disparities: low- and middle-income countries remain underrepresented, partly due to cost barriers and limited access to device technology. This imbalance suggests that the current knowledge base may disproportionately reflect high-resource settings, underscoring the need for more inclusive global research efforts. This geographic and demographic imbalance further underscores structural inequities in research participation, particularly for low- and middle-income countries and pediatric populations. Such underrepresentation likely arises from unequal research infrastructure, funding opportunities, and access to advanced devices rather than a lack of scientific engagement. Recognizing these disparities is crucial for promoting more equitable research policies, supporting global capacity-building, and ensuring that emerging technologies address the needs of diverse patient populations.

*Heart Rhythm* ranked as the most productive journal, while *Europace* was the most frequently co-cited, indicating their central roles in shaping discourse around CIEDs. The discrepancy between Heart Rhythm and Europace underscores the distinction between productivity and intellectual influence. Heart Rhythm functions as the primary outlet for high-volume, often industry-linked studies, whereas Europace exerts outsized influence through consensus statements, guidelines, and multicenter research that anchor clinical practice. However, both journals reveal a relative scarcity of pediatric and congenital device research, suggesting that influential discourse in the field continues to prioritize adult populations while leaving critical subgroups underrepresented. This dynamic highlights how bibliometric output alone does not equate to centrality in the field’s intellectual structure. Other influential journals include *PACE—Pacing and Clinical Electrophysiology*, *Journal of Cardiovascular Electrophysiology*, and *JACC: Cardiovascular Interventions*.

Among individual contributors, Kurt Stromberg (Medtronic Inc.) was the most productive and most co-cited author, with 20 publications (0.75%), 693 citations, and a total link strength (TLS) of 1273—highlighting the critical impact of industry leadership in device innovation. The prominence of Kurt Stromberg, an industry-affiliated author from Medtronic, underscores the pivotal role of device manufacturers in shaping the research landscape. His central position in both productivity and co-citation networks reflects how industry-driven innovation often sets research agendas, driving not only technical development but also influencing academic collaborations and citation patterns. This highlights the bidirectional nature of cardiac device research, where industry leadership does not merely contribute products but also actively shapes the intellectual architecture of the field.

The prominence of authors affiliated with major device manufacturers illustrates the pivotal role of industry in driving cardiac device innovation. Industry–academic partnerships have been essential to the rapid translation of novel pacing and defibrillation technologies into clinical use; however, this interdependence can also shape research priorities, funding distribution, and access to proprietary data, potentially amplifying commercially aligned agendas. Recognizing these dynamics is important when interpreting authorship networks and citation patterns, as they reflect both scientific productivity and the commercial context that influences the evolution of the evidence base.

### 4.2. Research HotSpots and Trends

Based on the analysis of the co-cited reference network, which is demonstrated in Figure 7, there are three research hotspots in the pacemaker field: use of leadless pacemakers (Cluster 1, red zone), permanent pacemakers on TAVR patients (Cluster 2, green zone), and the utility of self-powered pacemakers (Cluster 3, blue zone).

#### 4.2.1. Use of Leadless Pacemaker (Cluster 1)

“Use of leadless pacemaker” (Cluster 1) emerged as the most published topic, reflecting growing clinical interest in their efficacy, particularly in high-risk groups such as patients with prior device infections or limited venous access. Recent studies have highlighted their safety, reduced complication rates, and expanding functionality, including AV-synchronous and dual-chamber capabilities. Leadless pacemakers—self-contained, single-chamber devices implanted directly in the heart—have advanced rapidly. Their obvious advantage is the elimination of leads and surgical pockets, which has dramatically reduced infection risks. In the Micra post-approval registry, patients who had a prior pacemaker or defibrillator infection were given a Micra leadless pacemaker after system extraction. El-Chami et al. found no recurrent infections requiring device removal in these cases [21]. This suggests that leadless pacing is a safe alternative in patients with cardiac device infections, as it eliminates the need for subcutaneous generator pockets and transvenous leads—thereby reducing potential surfaces for bacterial colonization and limiting entry routes for infection.

In a large, contemporaneous Medicare cohort study, patients implanted with leadless VVI pacemakers had a 3.2% 6-month complication rate compared with 4.1% in those with transvenous VVI pacemakers. This corresponded to a 23% relative risk reduction in overall complications (95% CI, 4% to 38%). Device-related complications were also lower: 1.7% in the leadless group vs. 3.3% in the transvenous group, with a 49% relative risk reduction (95% CI, 33% to 61%) [33]. Patients implanted with leadless devices had lower rates of device revision and fewer lead- or pocket-related complications. Notably, they were more likely to have end-stage kidney disease and higher comorbidity scores compared to those receiving transvenous systems [33]. At 2-year follow-up, leadless-VVI pacemakers were associated with significantly fewer chronic complications and device-related reinterventions compared to transvenous-VVI systems in several high-risk subgroups, including patients with diabetes, COPD, malignancy, and tricuspid valve disease [43]. These findings suggest that eliminating transvenous leads and subcutaneous pockets may translate into improved long-term outcomes, enhancing both device safety and durability, particularly in patients most vulnerable to pacing-related complications.

Leadless pacemakers are also being refined to provide more advanced pacing modes. Originally, leadless devices only offered VVI (ventricular demand) pacing—pacing the ventricle alone. Recent studies have shown it is possible to achieve atrioventricular (AV) synchrony with a leadless system. The MARVEL 2 trial tested a downloaded accelerometer-based algorithm in Micra leadless pacemakers to sense atrial contractions via subtle accelerations (13). Steinwender et al. reported that this enhanced algorithm enabled the leadless pacemaker to track atrial activity and pace the ventricle in sync, effectively providing AV-synchronous pacing in patients with heart block. In this study, the majority of patients with complete AV block experienced restored AV synchrony when the algorithm was active, with corresponding improvements in cardiac output and functional status (since the pacemaker could coordinate with the atrial rhythm). This development marks an important step toward dual-chamber functionality in a single leadless device [13].

Even more impressively, the first dual-chamber leadless pacemaker system has now been realized. In 2023, Knops et al. reported a dual-chamber leadless pacing setup consisting of two wireless pacemaker capsules—one in the right atrium and one in the right ventricle—that communicate with each other. In an initial trial (Aveir DR i2i study), this system met its safety endpoints and demonstrated reliable atrial pacing with AV synchrony maintained over 3 months [38].

One question has been whether a device implanted directly inside the heart—like a leadless pacemaker in the right ventricle—might impair cardiac valve function. A 2019 prospective study by Beurskens et al. evaluated cardiac structure and function in patients with leadless pacemakers (Nanostim or Micra) over 12 months and found a significant increase in tricuspid regurgitation in 43% of patients. However, this degree of worsening was not significantly different from that observed in a matched cohort of patients with conventional dual-chamber transvenous pacemakers (38%; *p* = 0.395) [29]. This suggests that leadless pacing does not uniquely worsen tricuspid valve function compared to traditional leads. The study further identified that a more septal position of the leadless device was associated with increased TR severity, implicating mechanical interference with the tricuspid apparatus as a key contributor. Notably, leadless pacing was also associated with modest declines in right and left ventricular function and increased mitral regurgitation, likely due to pacing-induced dyssynchrony. Overall, these findings support the relative safety of leadless pacing regarding valvular effects but underscore the need for continued surveillance as these devices gain broader use [29].

Leadless pacemakers have proven especially advantageous in hemodialysis patients, who often face limited venous access and a high risk of infection. A dedicated multicenter analysis from the Micra investigational study reported that implantation in this group was generally quick (mean time ~27 min) and feasible, with no device-related infections observed at a mean 6-month follow-up. Although the overall complication rate was slightly higher than in non-dialysis patients, the safety profile was considered acceptable, reinforcing the value of leadless pacing in this high-risk population [44].

#### 4.2.2. Permanent Pacemaker on TAVR Patients (Cluster 2)

The intersection of pacemakers with structural heart interventions has been a hot topic, particularly the issue of conduction block after transcatheter aortic valve replacement (TAVR). TAVR can often injure the AV node or bundle branches (due to the proximity of the aortic valve to the conduction system), leading to high rates of new pacemaker implantation post-procedure. Multiple studies have characterized and tried to reduce this complication. A comprehensive 2021 review by Sammour et al. highlighted that conduction abnormalities requiring permanent pacemaker implantation remain common after transcatheter aortic valve replacement (TAVR), primarily due to mechanical injury to the conduction system located near the aortic root. Reported pacemaker implantation rates vary widely, ranging from 2.3% to 36.1% depending on the valve type and generation. The review emphasized that self-expanding valves (such as CoreValve and Evolut R) are associated with higher pacemaker rates—up to 37.7%—while balloon-expandable valves (such as SAPIEN 3) tend to have lower rates, reported as low as 4% in some studies. A consistently strong predictor of pacemaker requirement is pre-existing right bundle branch block (RBBB), which markedly increases the risk of high-grade atrioventricular block post-TAVR [15]. Indeed, a risk score (the Emory risk score) was developed by Kiani et al. to predict new pacemaker need after TAVR: patients with RBBB, longer baseline QRS duration, a history of syncope, and larger valve oversizing were at significantly higher risk [31]. This scoring system can help identify patients who might benefit from closer monitoring or preventive measures during TAVR.

To mitigate pacemaker rates, procedural techniques have been refined. Jilaihawi et al. introduced the “*MIDAS*” *approach* with repositionable self-expanding TAVR valves, aiming to minimize valve depth in the ventricular septum. By deploying the valve more precisely (higher relative to the conduction tissue), they were able to significantly reduce the incidence of new pacemakers compared to conventional implantation depths [8]. The concept is that a higher implant leaves the His bundle and left bundle branch area less compressed. Likewise, Sammour et al. showed that a systematic high implant technique with the SAPIEN-3 valve led to much shorter valve intrusion into the LV outflow tract, which lowered the rate of AV block and permanent pacemaker implantation [22].

Even when pacemakers are implanted after TAVR, not all patients end up truly needing lifelong pacing. Costa et al. examined pacemaker dependency one year after TAVR. They found that among TAVR recipients who received a pacemaker, only about 33–36% were pacing-dependent at 12 months (meaning two-thirds had recovered sufficient AV conduction not to require pacing) [27].

This raises an important question: might some pacemakers be placed “just in case” but ultimately prove unnecessary? It suggests a need for careful follow-up and re-evaluation of device indication in TAVR patients. In this study, patients with persistent high-grade AV block after 48 h were considered for permanent pacing, and electrophysiologic studies were used in selected cases to guide decisions. Ongoing research is evaluating strategies to minimize unnecessary pacemaker implantation after TAVR, since a device that is not truly needed may carry lifelong management burdens and potential risks without corresponding clinical benefit [27,45].

#### 4.2.3. Utility of Self-Powered Pacemaker (Cluster 3)

A major focus of recent research is eliminating the pacemaker’s battery limitations by harvesting energy from the body. Ouyang et al. demonstrated a “symbiotic” cardiac pacemaker that harvests biomechanical energy from cardiac motion. In a swine model, their self-powered pacemaker corrected sinus arrhythmia using only the heart’s natural movement as an energy source [7]. Similarly, Li et al. developed an implantable piezoelectric energy generator (iPEG) with a flexible skeleton and piezoelectric composites that harvested biomechanical energy from cardiac motion. When implanted at the apex of the porcine heart, the device generated sufficient output to directly power a modern, full-function pacemaker in vivo without an external battery [14]. This was a key step toward a truly self-powered pacemaker, addressing the traditional 5–12 year battery lifespan of implants.

Researchers have explored various energy-harvesting mechanisms. Ryu et al. integrated a coin-sized triboelectric nanogenerator (I-TENG) with a pacemaker to create a self-rechargeable system. Their device converts body motion and gravity into electricity (~4.9 μW/cm^3^) and successfully charged a small battery while maintaining ventricular pacing and sensing. This proof-of-concept shows that mechanical energy (from respiration, heartbeat, or movement) can continuously recharge an implant [10]. In another approach, Azimi et al. developed a flexible, biocompatible piezoelectric nanogenerator (PNG) based on electrospun PVDF fibers doped with ZnO and reduced graphene oxide (rGO). Implanted on the lateral wall of the left ventricle in a dog model, the device harvested up to 0.487 μJ per heartbeat—sufficient to power a commercial cardiac pacemaker [16]. Although the device was not directly integrated to pace the heart, the study demonstrated the feasibility of using lead-free polymer-based nanogenerators for self-powered cardiac implants.

Liu et al. developed a sandwiched wireless power transfer system using a bilateral coil receiver and a 160 kHz transmitter to recharge implantable pacemaker batteries. In bench experiments, the system delivered up to 5 W of power with transmission efficiency reaching ~88% [17]. This system’s robust coupling and flexible distance alignment would allow efficient transcutaneous charging without the need for surgeries to replace batteries.

Beyond permanent devices, researchers are even targeting temporary pacemakers used after cardiac surgery. Choi et al. reported a fully implantable, leadless, battery-free pacemaker that is bioresorbable [9]. Their thin, flexible device is powered by wireless RF energy (inductive coupling) instead of a battery. It provides pacing support for a programmed duration (days to weeks) and then dissolves harmlessly in the body. In animal models (rat, rabbit, canine) and human heart tissue, this bioresorbable pacemaker captured and maintained rhythm effectively, then completely bio-degraded after the temporary pacing need passed [9]. Such technology could prevent infections and complications associated with externalized leads in current temporary pacing systems.

The pursuit of self-powered pacemakers represents a transformative shift in cardiac device research, aiming to overcome the limitations imposed by finite battery life and repeated surgical replacements. By leveraging biomechanical, triboelectric, piezoelectric, and wireless power transfer technologies, recent studies have demonstrated the feasibility of harvesting energy directly from physiological motion or recharging devices transcutaneously. This growing trend reflects not only a technological evolution but also a clinical imperative—enhancing device longevity, reducing patient morbidity, and paving the way for fully autonomous, maintenance-free cardiac implants.

Notably absent from these clusters are studies addressing sex-based disparities in device implantation and outcomes, a gap that bibliometric mapping makes visible but that has yet to be systematically addressed. Given the well-documented sex differences in arrhythmia presentation and device benefit, this underrepresentation warrants closer attention in future research agendas.

The clusters identified in this study represent the principal research themes captured by our bibliometric analysis and reflect the established intellectual structure of the field. Nevertheless, it is important to recognize that, in contemporary clinical and research practice, certain topics—particularly conduction system pacing (CSP), encompassing His-bundle and left bundle branch area pacing—are gaining increasing attention and momentum. Such emerging trends may not yet be fully visible within our analysis window, as their citation growth remains in an early phase. This potential underrepresentation likely reflects the device-focused nature of our query and the emphasis on the top-cited corpus, both of which introduce standard bibliometric effects. Specifically, query-driven vocabulary bias arises when search terms favor certain conceptual domains—in this case, device terminology over pacing strategies—while citation-window bias occurs because citation-based selection inherently amplifies topics with a longer period of citation accumulation. As a result, CSP terminology may appear less prominent within the keyword network despite its current clinical relevance. Future bibliometric analyses using strategy-focused search terms could therefore complement our findings and capture these rapidly evolving areas with greater precision.

### 4.3. Strengths and Limitations

This bibliometric analysis provides a comprehensive overview of global research trends in cardiac devices, offering valuable insights into key contributors, publication patterns, and emerging research hotspots. By utilizing data from WoSCC, we ensured a systematic and high-quality literature analysis, capturing the most impactful studies in the field. The study also highlights institutional and author collaborations, journal influence, and keyword trends, making it a useful resource for researchers, clinicians, and policymakers interested in cardiac device innovations. The integration of visualized network analysis further enhances the clarity of knowledge structures and research evolution.

However, several limitations should be acknowledged. The study is restricted to English-language publications, which may exclude relevant findings from non-English sources. Additionally, the reliance on a single database (WoSCC) might introduce selection bias, as other databases such as Scopus or PubMed were not included. Citation-based metrics do not always reflect true clinical impact, as newer but highly significant studies may not yet have accumulated sufficient citations. Furthermore, co-citation and keyword analyses depend on available metadata, which may not fully capture the complexity of interdisciplinary research. Moreover, an inherent limitation of bibliometric design is that citation metrics, although robust indicators of academic visibility and influence, do not directly reflect scientific quality or clinical applicability. Citations capture patterns of recognition and dissemination within the scientific community, and thus primarily measure research influence rather than methodological rigor or translational impact.

It should also be recognized that the COVID-19 pandemic period substantially influenced scientific publication and citation patterns across biomedical research. Rapid dissemination of clinical guidance increased open-access availability, and intensified research collaboration temporarily altered citation velocity and network connectivity in many fields. The bibliometric structure observed in this study may therefore partly reflect these short-term fluctuations, especially in clinically oriented and device-related literature. Although our analysis was not temporally stratified, the pandemic context should be borne in mind when interpreting citation trends.

Lastly, our device-focused query and top-cited inclusion potentially introduce query-driven vocabulary bias and citation-window effects, which may under-represent rapidly evolving pacing strategies such as CSP; this should be considered when interpreting keyword-level signals.

## 5. Conclusions

This bibliometric analysis offers a panoramic view of the research landscape surrounding cardiac devices from 2019 to 2024, illuminating key contributors, institutional collaborations, influential journals, and prevailing thematic clusters. The findings highlight an expanding global interest, with the United States maintaining a dominant role in both output and citation influence, supported by strong academic–industry partnerships. Leadless pacemakers, pacemaker use following TAVR, and the emergence of self-powered pacing technologies have emerged as major research hotspots, reflecting the field’s dynamic response to both clinical challenges and technological opportunities.

Through citation mapping and co-authorship networks, this study identifies not only the volume but also the impact and connectivity of research contributions. It underscores a field marked by rapid innovation, translational efforts, and multidisciplinary integration. As cardiac device technologies continue to evolve—toward miniaturization, battery-free operation, and individualized therapy—the bibliometric trends observed herein provide a critical foundation for future investigations and innovation strategies. Continued bibliometric surveillance will be essential to track how scientific priorities shift and how novel therapies are integrated into clinical practice.

## Figures and Tables

**Table 1:** Top Ten Productive Countries.

Rank	Country	Publications	Percentage	Citations	Citation per Publications	Total Link Strength
1	USA	362	33%	7198	19.9	414
2	Germany	128	11.9%	2200	17.2	289
3	Italy	111	10.4%	1776	16.0	243
4	China	103	9.6%	2677	26.0	82
5	England	89	8.3%	1646	18.5	256
6	Netherlands	76	7.1%	1312	17.3	209
7	France	70	6.5%	1382	19.7	197
8	Japan	66	6.2%	919	13.9	79
9	Spain	66	6.2%	1284	19.5	195
10	Canada	59	5.5%	983	16.7	149
Total		1071	100	20,394		1964

**Table 2:** Top Ten Productive Institutions.

Rank	Organization	Documents	Percentage (%)	Citations	Citation per Publications	Total Link Strength
1	Emory Univ	29	16.66	832	4.78	451
2	Mayo Clin	21	12.06	318	1.82	195
3	Duke Univ	19	10.91	662	3.80	373
4	Karolinska Inst	19	10.91	375	2.15	37
5	Univ Amsterdam	18	10.34	338	1.94	113
6	Cleveland Clin	15	8.62	358	2.05	88
7	Medtronic Inc	15	8.62	374	2.14	276
8	Duke Clin Res Inst	14	8.04	473	2.71	334
9	Columbia Univ	12	6.89	288	1.65	42
10	Univ Leipzig	12	6.89	135	0.77	33
Total		174	99.94	4153	23.81	1942

**Table 3:** Top Ten Journals according to number of publication/Co-citation.

Rank (Based on Number of Publication)	Journal Name	Publications	Percentage (%)	Total Link Strength	Rank (Based on Number of Citation)	Journal Name	Citations	Citation Per Publications	Total Link Strength
1	heart rhythm	53	19.202	132	1	europace	891	22.84	79
2	journal of cardiovascular electrophysiology	51	18.47	108	2	heart rhythm	820	15.47	132
3	pace—pacing and clinical electrophysiology	40	14.49	81	3	jacc—cardiovascular interventions	655	59.54	65
4	europace	39	14.13	79	4	journal of cardiovascular electrophysiology	623	12.21	108
5	journal of interventional cardiac electrophysiology	23	8.33	51	5	pace—pacing and clinical electrophysiology	421	10.52	81
6	scientific reports	14	5.07	14	6	jacc—clinical electrophysiology	398	28.42	55
7	resuscitation	14	5.07	6	7	european heart journal	337	48.14	12
8	journal of the american heart association	14	5.07	54	8	journal of the american heart association	275	19.64	54
9	jacc—clinical electrophysiology	14	5.07	55	9	circulation-arrhythmia and electrophysiology	256	25.6	25
10	international journal of cardiology	14	5.07	23	10	journal of interventional cardiac electrophysiology	214	9.3	51
Total		276	99.972						

**Table 4:** Top Ten Co-Cited authors.

Top 10 Publishing Authors					Top 10 Cited Authors			
Rank	Author	Publications	Percentage (%)	TLS	Rank	Author	Citations	Total Link Strength
1	stromberg, kurt	20	15.4%	1273	1	stromberg, kurt	693	1273
2	el-chami, mikhael f.	18	13.8%	1074	2	li, ning	612	73
3	piccini, jonathan p.	17	13.1%	1096	3	zhang, hao	594	72
4	garweg, christophe	14	10.8%	695	4	xie, feng	587	67
5	roberts, paul r.	13	10.0%	721	5	ma, ye	574	64
6	clementy, nicolas	10	7.7%	398	6	piccini, jonathan p.	569	1096
7	epstein, laurence m.	10	7.7%	528	7	el-chami, mikhael f.	487	1074
8	knops, reinoud e.	10	7.7%	290	8	garweg, christophe	348	695
9	boriani, giuseppe	9	6.9%	69	9	epstein, laurence m.	331	528
10	cha, yong-mei	9	6.9%	354	10	roberts, paul r.	321	721
Total		130	100%	6498	Total		5116	0

**Table 5:** Top Ten Funding Agencies.

Rank	Funding Institution	Country	Number of Articles	Percentage
1	National Institutes of Health NIH USA	USA	127	23.69%
2	United States Department of Health Human Services	USA	127	23.69%
3	National Natural Science Foundation of China NSFC	China	66	12.31%
4	Medtronic	USA	49	9.14%
5	NIH National Heart Lung Blood Institute NHLBI	USA	35	6.53%
6	Boston Scientific	USA	29	5.41%
7	German Research Foundation DFG	Germany	28	5.22%
8	American Heart Association	USA	25	4.66%
9	Japan Society for The Promotion of Science	Japan	25	4.66%
10	Ministry Of Education Culture Sports Science And Technology Japan MEXT	Japan	25	4.66%

**Table 6:** 35 Most Cited References.

Rank	Article	Citations	Total Link Strength
1	Ouyang (2019) [7]	446	5
2	Jilaihawi (2019) [8]	203	11
3	Choi (2021) [9]	194	2
4	Ryu (2021) [10]	187	3
5	Gutruf (2019) [11]	152	2
6	Chen (2019) [12]	148	1
7	Steinwender (2020) [13]	141	10
8	Li (2019a) [14]	128	6
9	Sammour (2021a) [15]	124	12
10	Azimi (2021) [16]	122	3
11	Liu (2019) [17]	114	0
12	Hastings (2019) [18]	108	2
13	Shah (2019) [19]	105	5
14	Burri (2021) [20]	101	2
15	El-chami (2019a) [21]	92	10
16	Sammour (2021b) [22]	83	4
17	Van der werf (2019) [23]	81	0
18	El-chami (2020a) [24]	79	9
19	Miften (2019) [25]	78	0
20	Jukema (2019) [26]	77	0
21	Costa (2019a) [27]	72	13
22	Amaya (2019) [28]	69	0
23	Beurskens (2019a) [29]	69	4
24	Rohde (2020) [30]	66	1
25	Kiani (2019) [31]	65	6
26	Funk (2019) [32]	65	0
27	Piccini (2021) [33]	64	10
28	Javier Garcia-Fernandez (2019) [34]	64	0
29	Liang (2019) [35]	64	1
30	Bychkov (2020) [36]	63	3
31	Kazmirczak (2019) [37]	63	1
32	Knops (2023) [38]	62	3
33	Han (2022) [39]	62	3
34	Haunreiter (2019) [40]	61	0
35	El-chami (2019b) [41]	61	6

**Table 7:** Top Ten Related Keywords.

Rank	Keyword	Occurrences	Percentage (%)	Total Link Strength
1	Pacemaker	205	35.5	243
2	Leadless Pacemaker	77	13.3	92
3	Heart Failure	51	8.8	93
4	Transcatheter Aortic Valve Replacement	45	7.8	81
5	Implantable Cardioverter-defibrillator	42	7.3	73
6	Cardiac Resynchronization Therapy	36	6.2	77
7	Atrioventricular Block	32	5.5	51
8	Atrial Fibrillation	31	5.4	50
9	Permanent Pacemaker	29	5.0	37
10	Sudden Cardiac Death	29	5.0	50
Total		577	100	847

## Data Availability

Data were obtained from Clarivate’s Web of Science Core Collection (WoSCC) on 27 November 2024 (coverage: 2019–2024; query: “cardiac defibrillator” OR “pacemaker”). Because WoSCC is a subscription-based proprietary database, the raw exported records cannot be publicly deposited. The complete search strategy—including search terms, filters, and date of retrieval—is provided in the Methods section. A curated dataset containing all included records (titles, authors, publication details, and citation metadata) is available from the corresponding author (M.D.A.S.) upon reasonable request. The R scripts, VOSviewer map files, and data-cleaning code used in the analysis are also available upon reasonable request.
